# Training sensory signal-to-noise resolution in children with ADHD in a global mental health setting

**DOI:** 10.1038/tp.2016.45

**Published:** 2016-04-12

**Authors:** J Mishra, R Sagar, A A Joseph, A Gazzaley, M M Merzenich

**Affiliations:** 1 Sandler Neurosciences Center, Department of Neurology, University of California, San Francisco, San Francisco, CA, USA; 2 Department of Psychiatry, University of California, San Francisco, San Francisco, CA, USA; 3 Department of Psychiatry, All India Institute of Medical Sciences, New Delhi, India; 4 Department of Physiology, University of California, San Francisco, San Francisco, CA, USA; 5 Posit Science Corporation, San Francisco, CA, USA

## Abstract

Children with attention deficit/hyperactivity disorder (ADHD) have impaired focus on goal-relevant signals and fail to suppress goal-irrelevant distractions. To address both these issues, we developed a novel neuroplasticity-based training program that adaptively trains the resolution of challenging sensory signals and the suppression of progressively more challenging distractions. We evaluated this sensory signal-to-noise resolution training in a small sample, global mental health study in Indian children with ADHD. The children trained for 30 h over 6 months in a double-blind, randomized controlled trial. Training completers showed steady and significant improvements in ADHD-associated behaviors from baseline to post training relative to controls, and benefits sustained in a 6-month follow-up. Post-training cognitive assessments showed significant positive results for response inhibition and Stroop interference tests in training completers vs controls, while measures of sustained attention and short-term memory showed nonsignificant improvement trends. Further, training-driven improvements in distractor suppression correlated with the improved ADHD symptoms. This initial study suggests utility of signal-to-noise resolution training for children with ADHD; it emphasizes the need for further research on this intervention and substantially informs the design of a larger trial.

## Introduction

It is estimated that nearly 10% of children worldwide are diagnosed with attention deficit/hyperactivity disorder (ADHD).^1, 2, 3^ Children with ADHD manifest poor neurocognitive function in daily life activities that require attention, memory and goal-management skills.^4, 5^ These problems put them at risk for failure and dropout from school, increase susceptibility to addiction disorders and criminality, and foretell poorer quality of life.^6, 7, 8, 9^ Given the large socioeconomic and health-care burden posed by ADHD, the development of effective and scalable therapeutics for impaired children has great practical relevance.

Stimulant medications for ADHD (methylphenidate and amphetamine) are the standard of care for treatment, now prescribed for 3.5 million American children.^10^ Although they show high efficacy in mitigating ADHD symptoms in the short term (2–3 months), limitations include minimal long-term benefit, nonselective amplification of neurocognitive function and accumulation of drug-related side effects.^11, 12, 13^ Parents prefer to opt for non-pharmacological therapies, but none have shown significant efficacy in blinded controlled trials.^14, 15^ Thus, the need for scientifically informed therapeutics for children with ADHD remains unfulfilled.

Several decades of neuroscience research have shown that the brain is plastic and adaptive to experience.^16^ Further, neuroplasticity-targeted training programs have been successfully created to improve the attention and learning outcomes in children.^17, 18^ Principally, these studies implement performance-adaptive attended-signal training, that is, they challenge the user to attend to progressively more difficult and subtle sensory signals. However, a main limitation of this research has been that it does not impact the neurocognitive response to unattended, distracting information. This is a problem because there is much evidence showing that children with ADHD have heightened distractibility,^19, 20, 21, 22^ which is unaltered or even exacerbated by medications.^23, 24^

To address this issue, we recently engineered neuroplasticity-targeted training in parallel animal and human experiments, which specifically suppresses neural responses to distractions and reduces distractor-related, false positive errors in behavior.^25^ We then combined the attended-signal training and distractor-suppression training into an integrated training program: ‘ONTRAC' (acronym for Online Neuroplasticity Targeted Remediation of Attention deficits in Children^26^), and hypothesized that ONTRAC would impart robust and sustainable benefit for children with ADHD.

**Figure 1:**
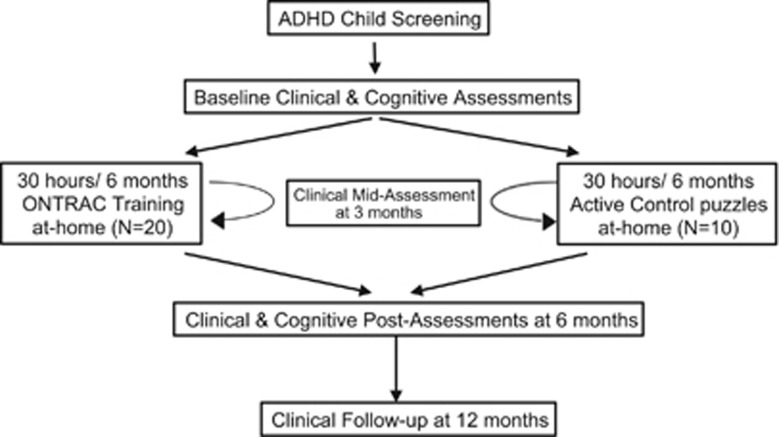
Study design for the ONTRAC double-blind randomized controlled trial (ClinicalTrials.gov as NCT01772485). ADHD, attention deficit/hyperactivity disorder; ONTRAC, Online Neuroplasticity Targeted Remediation of Attention deficits in Children.

In an Indo-US global mental health collaboration, we evaluated ONTRAC in Indian children with ADHD using a double-blind randomized controlled study design (Figure 1). Participants in the study were randomized to either the ONTRAC training group, or to a matched, active control group that played nontherapeutic video games. Both the groups were provided access to their respective programs for 30 h over a period of up to 6 months of training. All performance and logged-activity were monitored remotely online. In all children, parent-based ratings of ADHD symptom severity formed the primary clinical outcome measure and were assessed at baseline, mid-training, post-training and at a 6-month follow-up visit. The follow-up visit assessed the sustainability of training outcomes after a 6-month period during which participants had no access to ONTRAC or control computer games. The study also incorporated objective tests of cognitive function at baseline and post-training in all children; these tests included measures of sustained attention, response inhibition, short-term memory and the Stroop interference task. This initial study was, thus, designed to measure the magnitude of training-related cognitive and behavioral improvements in children with ADHD, to determine whether ONTRAC use is practical in a low-to-middle-income country, and to determine the sustained behavioral impacts of this training in a clinical ADHD population.

## Materials and Methods

This study was registered on ClinicalTrials.gov before initiation (NCT01772485).^27^

### Participants

Thirty-one children with ADHD (mean age 12±1.9 years, two females) participated in the study; 30 participants were originally planned for enrollment over a 2-year research period estimated based on the monthly frequency of ADHD referrals to the clinic. One parent for each child gave written informed consent and the child provided verbal consent for the study in accordance with the guidelines set by the Institutional Ethics Committee at the All India Institute of Medical Sciences (AIIMS), New Delhi, India. The study was also approved by the Health Ministry Screening Committee (HMSC), which oversees all international collaborative medical research in India. Enrollment occurred over a 1.5-year period exclusively based on incoming referrals to the hospital's child psychiatry clinic from the local community in New Delhi. Study participation was monetarily compensated at the standard rate used in clinical psychiatry studies at AIIMS.

Thirty healthy children, with no ADHD or other neuropsychiatric disorder, also made a single clinic visit and parents provided ratings of ADHD-associated behaviors on the ADHD rating scale (RS IV^28^) confirming non-ADHD status. The same consent, screening and compensation procedures were used for ADHD and non-ADHD children.

### Screening

The children were screened to meet the inclusion/exclusion criteria of the study. For all children, ADHD diagnosis was verified as per US standards; a structured clinical interview was conducted using the Schedule for Affective Disorders and Schizophrenia for School-Age Children (K-SADS-PL)^29^ and the SWAN ADHD rating scale was completed by a parent (cutoff >2.11 for ADHD-combined type, >2.48 for ADHD-inattentive type, >2.00 for ADHD-hyperactive type).^30^ Inclusion criteria also required fluency in English, IQ>80 as assessed by the Indian version of the Weschler Intelligence Scale for Children,^31^ and access to an Internet-connected computer/laptop at or near home. Children on psychostimulant medications were not excluded from the study. Fifteen of 31 children with ADHD were on stimulant medications; type and dosage of all medications were recorded and children were not allowed to change their medication dosage during the course of the study. Exclusion criteria were fulfilling criteria for diagnosis of clinically significant oppositional defiant disorder, autistic or Asperger's syndrome, depression, history of seizure disorder or seizure episodes over the last 2 years, and motor/ perceptual handicap that prevented computer use.

### Assessments

The study included a training feasibility survey that was administered in all participants post intervention; the participants responded on a seven-point Likert scale to the survey questions (Table 1). To determine initial efficacy, we included clinical and cognitive assessments as follows:

The ADHD rating scale (RS IV) as completed by the parent served as the primary clinical behavioral outcome measure of ADHD symptom severity.^28^ This measure was completed by the parent at the clinic at four time points: baseline, mid-intervention, post-intervention and at a 6-month follow-up. The children did not access their assigned intervention after the post-intervention assessment; hence the follow-up visit assessed sustainability of outcomes in the absence of the intervention.

Clinical Global Impression (CGI)—severity of illness scale^32^ was administered by the clinician as a secondary outcome measure at baseline, mid-intervention, post-intervention and at the 6-month follow-up.

Cognitive assessments were conducted at baseline and post-intervention. These included the following tests:

(i) Sustained attention: the test of variables of attention is a 20-min test of which the first 10 min of testing is used to measure sustained attention.^33^ During this time, the participant is presented with visual target and non-target stimuli, which are square stimuli appearing in the upper or lower visual field, respectively. Visual targets occur infrequently and are randomized on 22.5% of trials. The participant detects targets with a spacebar response and withholds responses to non-targets. This test challenges selective sustained attention as it requires accurate responding to infrequent targets. The signal detection sensitivity index (*d*′) was evaluated as the performance metric for this test.^34^

(ii) Response inhibition: the second half of the test of variables of attention provides a measure of response inhibition. Like the sustained attention test, this test is also composed of visual target and non-target stimuli, squares in the upper and lower visual field, respectively. In this test, targets occur frequently on 77.5% of trials and are detected with a response on the spacebar. Responses to non-target stimuli must be withheld. This test measures response inhibition as it challenges the user to infrequently withhold a response to the non-target stimuli. The signal detection sensitivity index (*d*′) was evaluated as the performance metric for this test.^34^

(iii) Short-term memory span: short-term memory span measures the ability to maintain sequences of information in mind for brief periods of time. We measured this ability in the visuospatial domain using the spatial capacity task and in the verbal domain using the verbal capacity task.^35, 36^ In the spatial capacity task, participants are presented a test array of varying span loads, that is, one, three, five or seven yellow circles appearing for 2 s. The test array is followed by a 3-s delay and then a single green circle probe appears for 3 s. The probe circle matches the position of one of the test circles on 50% of trials or does not match any of the test circle positions on the other 50% trials. Match and non-match probe trials are randomized. The participants respond yes/no for perceived match/non-match between the probe circle and the test array. The verbal capacity task is set up identical to the spatial capacity task except that the test array consists of a sequence of alphabets of span length three, five, seven or nine. The probe in the verbal capacity task is a single letter that matches one of the letters in the test array on 50% of trials or does not match any of the test array letters on the other 50% trials. Again, the participants must respond yes or no as per their perceived match/non-match between the probe and the test array letters. For these tests, the accuracies were stable across assessment time points, hence the response speed specifically for high-span load trials (spatial capacity task: five and seven circles; verbal capacity task: seven and nine letters) was used as the performance metric.

(iv) Stroop interference test: the Stroop color and word interference test was used as a component measure of executive function.^37^ We used a non-computerized test version in which the participants are asked to read off three printed sheets with 100 stimuli on each sheet. The word sheet presents the words blue, green and red typed in black and white in a shuffled sequence, and the participant reads the word list. The color sheet presents ‘XXXX' in blue/green/red color and the participant reports the list of colors. The color–word sheet presents the words blue, green and red typed in interfering color ink, for example, the word blue typed in green ink, and the participant must report the color of the word but not read the word. The participant is given 45 s on each sheet to respond to as many stimuli as they can. The interference score is calculated as the difference between correctly responded items on the color–word sheet minus the number of correctly responded items on the color sheet. The raw interference score is a negative number and its magnitude represents the degree of interference; this was used as the Stroop task performance measure.

### Intervention arms

After screening and baseline clinical and cognitive assessments, the study participants were randomized to either the ONTRAC intervention arm (*N*=21) or an active control game playing arm (*N*=10). In this initial trial, randomization was weighted towards more participants in the ONTRAC group to minimize the effect of dropout or variable-training compliance on data analyses. On/off medication status was pseudo-randomized between the two arms to have approximately the same number of participants on vs off medications in each arm; for this, blocked randomization was followed in batches of eight participants at a time.

### Double blinding

The clinician assessors directly interacting with the participant were blind to the intervention assigned to the child throughout the study. A separate designated research personnel was in charge of randomization and provided the online website links and login information to either the ONTRAC or active control intervention; this personnel was not responsible for any assessments. Finally, the children and their families in either arm were only aware of the existence of their own intervention arm. As all the families were blind to the existence of the other study arm, the placebo effect was maximized in both the arms; this was further confirmed by similar ratings on the post-intervention feasibility survey in the ONTRAC vs control arm (Table 1).

The children in both study arms were asked to engage with their assigned intervention for 30 h in a recommended schedule of 30 min per session, three to five sessions per week, for a total of 60 sessions over a maximum duration of 24 weeks (6 months). All training was performed in the home setting. In rare cases, a technician visit was set up at the participant's home to help with technical troubleshooting, if necessary; this technician was blind to the goals of the study. To facilitate compliance, the participants in both groups received regular weekly email and phone reminders over the 6-month intervention period; the person providing the weekly reminders was blind as to which intervention, ONTRAC or control, was being tested as novel therapy. Compliance and performance were continuously monitored on an online data server. All participants were encouraged to complete their assigned training during the 6-month period, and were post-assessed at 6 months irrespective of how much training was actually completed.

### ONTRAC

This training consisted of 25 online cognitive exercise modules that either focused on attended-signal training or distractor-suppression training. In attended-signal training exercises, a target stimulus was detected in a sequence of constant background distractor stimuli.^38^ On correct identification, the salience of the target stimulus was adaptively reduced on the next trial to increase the challenge. Incorrect responses led to an increase in target salience or reduced challenge. In distractor-suppression training exercises, the target stimulus was held constant throughout the exercise and challenge was modulated by progressively increasing the similarity of the distractors to the target after correct trials or decreasing the similarity of the distractors to the target after incorrect trials.^25^ There were separate distractor training exercises that contained explicit distractors (that is, distractors that occurred during a pre-specified period during the trial and had to be completely ignored) or implicit distractors (that is, distractors that the participants had to perceive and determine as distractors). In all cases, adaptive progressions were managed using staircase algorithms^39^ that modulated challenge so as to maintain exercise difficulty in the 75–85% performance range, which was challenging yet not frustrating.

Of the 25 exercise modules, there were an equal number of (eight each) attended-signal training modules, explicit distractor training and implicit distractor training modules. In each set of eight modules, there were four modules each presenting visual and auditory stimuli. The visual modules progressed in stimulus complexity from (1) simple Gabor line orientation patches to (2) colored shapes and textures to (3) directional motion stimuli to (4) complex landscape scenes. Auditory modules progressed in stimulus complexity from (1) simple tonal stimuli to (2) frequency sound sweeps (both high-to-low and low-to-high frequency sweeps) to (3) phonemic sounds (‘ba', ‘da' and so on) to (4) similar sounding words. For these 24 exercises ((4 visual+4 auditory) × 3 (attended-signal training/explicit distractor training/implicit distractor training)), the response format on all trials was two-alternative forced choice, that is, respond ‘yes' if a target was detected else respond ‘no'. The twenty-fifth exercise was a visual exercise in which the participants practiced response inhibition, that is, withheld their response to specific visual target images that contained complex face and/or scene content, while responding to all other complex distractor images; challenge was adaptively increased in this exercise through faster interstimulus times.

The participants were presented up to seven exercises in each 30-min session, 3–5 min duration each. Exercises with simpler stimuli appeared earlier in training and complex stimuli exercises were unveiled after the simple stimuli exercises had been completed. Overall, these adaptive exercises systematically trained sustained attention in their continuous performance demands, exercised working memory in that specific target stimuli had to be maintained in memory over several trials amid distractors, and allowed practice on response inhibition and resolution of response conflict between two-alternative choices. Notably, however, these training exercises differed in stimulus content and task mechanics from the standard cognitive assessment tests performed at baseline and post-intervention.

**Figure 2:**
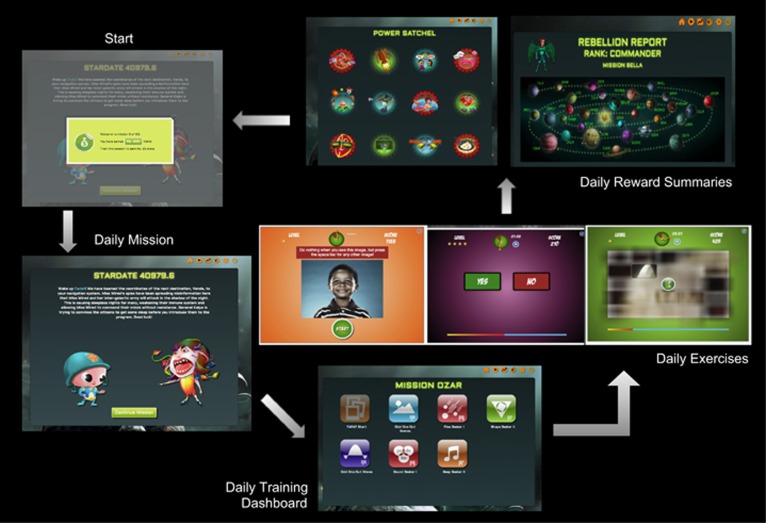
Flow through the ONTRAC program. The user enters the program (top right) and is provided a recap of accumulated rewards followed by a daily training mission. The user then continues to a dashboard of seven daily exercises that can be completed in a 30-min session. Daily exercise snapshots are shown. After session completion, the user's rank is updated along with progress through planetary missions and earned super powers. The user then returns to ‘Start' and accesses the next session. ONTRAC, Online Neuroplasticity Targeted Remediation of Attention deficits in Children.

To promote compliance, all the exercises were compiled in a meta-game wrapper in which the participant could choose a male or female avatar, who progressed through an intergalactic journey solving crises, fighting a villain, gaining super powers and advancing through performance ranks from cadet to commander; rank progression was based on engagement with the training program. Overall, the trainee was motivated with rewards and feedback at multiple levels: (1) on individual trials within each exercise game, (2) on a running scoreboard and star/trophy displays that updated on each trial and at the end of each exercise, and (3) daily performance summary graphs across exercises as well as graphics of the victories through the planetary journey and accumulated powers. The flow through this meta-game wrapper is shown in Figure 2.

### Active control

The children in this group were assigned to play Hoyle puzzle games, which have also been previously adopted as a control for cognitive training in a schizophrenia trial;^40^ these games do not target refinement of signal-to-noise resolution. Thirteen games from the Hoyle suite of games were chosen for this purpose, which included games like Checkers, Mazes, Solitaire, tile games and word games like Anagrams and Hangman. Access to these games was controlled by an online portal that monitored logged hours and also randomly selected four games (7-min each) that the child should play on any given day within a 30-min session. Matched to the ONTRAC group, the active control group also engaged in 30 h of computer game-based interaction and was contacted for compliance check-ins at the same weekly frequency. This group thus successfully controlled for placebo effects, practice effects on the repeated assessments, time spent engaging with a disciplined computer activity in the home setting, contact with research personnel and monetary payments. This active control suite of games was also selected to control for the nonspecific and nonadaptive engagement of attention and working memory systems, executive functions and motivation through the reinforcement of graphics-based computer games. We confirmed that the control group indeed had the expectation that their intervention was equally therapeutic and useful as the ONTRAC group, using the post-intervention feasibility survey (Table 1).

### Data analyses

Nonparametric tests were used for all scale-based responses, that is, for feasibility ratings, ADHD symptom severity ratings and CGI ratings. For these assessments, between-group differences were characterized using the Wald–Wolfowitz Runs test. The repeated-measures Friedman analysis of variance (ANOVA) was applied to assess within-group modulation of ratings over the multiple assessment time points, and the Kruskal–Wallis ANOVA was used to inspect whether medication status interacted with assessment outcomes. Within-group differences between baseline and a subsequent assessment were further characterized using Wilcoxon matched-pairs tests.

Parametric tests were used to assess baseline to post-intervention changes in cognitive outcomes. Parametric between-group testing was applied after confirming that between-group cognitive outcomes data were homoscedastic, that is, had equivalent variances as per the Levene's test. A repeated-measures ANOVA was conducted with training group and cognitive assessments as factors followed by *post hoc* between-group two-tailed *t*-tests.

The effect sizes were calculated using Cohen's *d* and corrected for small sample bias using the Hedges and Olkin approach.^41, 42^ Pearson product–moment correlation was used to assess the relationship between change in ADHD symptom severity and change in distractor errors with ONTRAC training.

## Results

### Program feasibility

The study was registered on ClinicalTrials.gov (NCT01772485) with planned enrollment for 30 participants.^27^ We enrolled 31 children from New Delhi diagnosed with ADHD (mean age 12±1.9 years, 2 females, 15 on medication). All children met study inclusion/exclusion; they had no psychiatric comorbidities, IQ>80, were fluent in English, and had access to a home computer with an Internet that could adequately support the web-based intervention. The children were randomized in a 2:1 ratio to ONTRAC vs the active control online computer games intervention and medication status was pseudo-randomized, such that there were 21 children assigned to ONTRAC (10 on medication) and 10 children assigned to control game-play (5 on medication).

Three participants (all on medication) randomized to the control group dropped out of the study after baseline assessments and before any intervention exposure. Twenty-one children continued through the study in the ONTRAC training group; seven children were retained in the control group (five off and two on medication). All children were assigned 30 h of their designated intervention to be completed over a 6-month period. All the children in the control group complied with this assignment. However, partial compliance was observed among the ONTRAC trainees. Eleven participants completed the full 30 h of training; 10 children engaged suboptimally, completing less than half of the full program hours (partial training range: 1–13 h, mean 4.5±3.6 h). Of the 11 ONTRAC completers, 6 were on medication; and of the 10 partial trainees, 4 were on medication; it did not appear that training adherence was influenced by medication status (logistic regression of training hours vs medication status in 21 ONTRAC participants: *R*=0.17, *P*=0.47).

When surveyed, the reasons for partial compliance included erratic Internet access, technical computer problems and unexpected home stressors during the study period including major illness in the family and change of residence. The families did not report any particular problems with the training program per se, but we acknowledge that 52% vs 70% program completion rates in the ONTRAC vs control group was aligned with our initial assumption that children may more easily adhere to the control video games that were inherently fun and engaging.

All participants completed feasibility surveys at the post-intervention visit (Table 1). ONTRAC completers vs partial trainees did not significantly differ on any of the feasibility metrics (all *P*>0.1); they found the training equally enjoyable, easy to understand, initiate, navigate, therapeutic and so on. Yet, we cannot rule out the possibility that ONTRAC completers vs partial trainees had inherently different motivation; although we did not assess this formally in the study, anecdotally the study personnel performing weekly check-ins observed that training completers possibly had more supportive family environments.

Comparing ONTRAC completers vs controls both of whom engaged in 30 h of intervention, the control group found their game-play more enjoyable (*P*=0.04), less frustrating (*P*=0.04), and easier to initiate (*P*=0.04) confirming our initial assumptions that control games may be more engaging (Table 1). Other feasibility aspects did not significantly differ between ONTRAC completers and controls.

### Initial program efficacy: ADHD behavioral symptoms

All assessment data were successfully obtained from intervention completers in the ONTRAC (*n*=11) and control groups (*n*=7). These included the primary outcome measure: parent-based ratings of ADHD symptom severity, a secondary non-ADHD-specific measure of CGI and standard cognitive tests. The ADHD severity ratings and CGI scales were measured at baseline, mid- and post-intervention and at a 6-month follow-up. Cognitive assessments included computer-based standard tests of sustained attention, response inhibition (that is, non-impulsivity), short-term memory and Stroop interference; these were obtained at baseline and post-intervention. ONTRAC partial trainees had missing data for three participants on CGI and cognitive assessments, and their sporadic program engagement (4.5±3.6 training hours) did not allow for interpretable comparisons with ONTRAC completers or controls for these assessments; however, partial trainee data were acquired and analyzed for change on the primary outcome measure of ADHD severity ratings.

**Figure 3:**
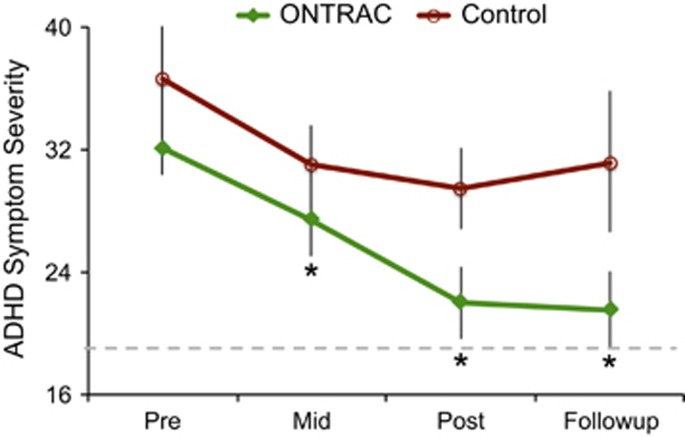
Parent-based ADHD symptom severity ratings at baseline (Pre), mid-intervention (Mid, 3 months from Pre), post-intervention (Post, 6 months from Pre) and at follow-up (12 months from Pre). The children did not access their assigned intervention between the post-assessment and follow-up. Data points are average ratings for ONTRAC completers (*n*=11, in green) vs active control participants (*n*=7, in red); error bars are standard errors (s.e.m). * indicates that only the ONTRAC group showed significant and sustained improvement from baseline. At follow-up, the ONTRAC scores did not significantly differ from the scores in healthy children (dashed gray line represents upper 95% confidence interval of healthy ratings). ADHD, attention deficit/hyperactivity disorder; ONTRAC, Online Neuroplasticity Targeted Remediation of Attention deficits in Children.

Figure 3 shows changes in the primary outcome: parent-based ratings of ADHD symptom severity for ONTRAC completers vs controls. At baseline, these groups did not significantly differ on ADHD symptom-severity ratings. The change in symptom severity for ONTRAC vs controls from baseline to mid-/post-intervention did not reach between-group significance (*P*>0.05), but was significant at follow-up (*P*=0.04). Within-group ANOVAs applied over the four assessment time points (baseline, mid-, post-, follow-up) showed that symptom severity ratings changed significantly in the ONTRAC group (*P*<0.003) but not in the control group (*P*<0.1). In addition, similar to the control group, partial trainees did not show change in symptom severity over the four assessments (*P*<0.08). These results suggested that full adherence with the ONTRAC program is important for improvements in ADHD severity. Of note, the outcomes for ONTRAC completers at the four assessment time points did not show an interaction with medication status (all *P*>0.27).

Within-group matched-pairs analyses of symptom severity ratings in the ONTRAC group identified that improvements from baseline occurred at all subsequently assessed time points (mid: *P*=0.03, post: *P*=0.005, follow-up: *P*=0.04), while these analyses in the control and partial training group found no significant results.

Preliminary effect sizes (Cohen's *d*, ONTRAC completers vs controls) were calculated correcting for small sample bias.^41, 42^ These effect sizes were, respectively, 0.31 at post-intervention and 0.36 at follow-up, highlighting the sustained gains of the ONTRAC intervention. These are clinically meaningful effect sizes; nevertheless, a larger, well-powered trial is needed to confirm these findings. Overall, the primary outcome measure in our study, that is, ADHD symptom severity, was successfully improved by completed ONTRAC training but not by the control intervention.

To ascertain the degree of normalization in ONTRAC completers on the primary outcome measure, we also obtained ADHD rating scale data from parents of 30 healthy children from the same community. The healthy children were age- and gender-matched and screened to ensure that they had no neuropsychiatric disorder. The comparison between ADHD ONTRAC completers (*n*=11) and healthy children (*n*=30) showed that the two cohorts significantly differed at baseline, mid- and post-intervention (all *P*<0.0005), but not at follow-up (*P*=0.06, Figure 3). This result suggests sustained benefit of the ONTRAC intervention towards normal behavior.

The CGI was a secondary measure in our study and provides a rough measure of the severity of mental illness on a 1–7 scale. Note that CGI is not specific to ADHD and the clinical assessor was blind to intervention assignment for the child. Nonparametric ANOVA over the four assessed time points did not find a significant effect in the ONTRAC group (*P*<0.09) but did find a significant modulation in the control group (*P*<0.007). However, further testing using Wilcoxon matched-pairs tests did not find significant changes from baseline in either group at any of the subsequent assessment time points (all *P*>0.07). It remains possible that as CGI is a generic clinical measure of mental illness, it may not have the sensitivity to capture outcomes specific to ADHD in this initial trial.

### Initial program efficacy: cognitive functions

**Figure 4:**
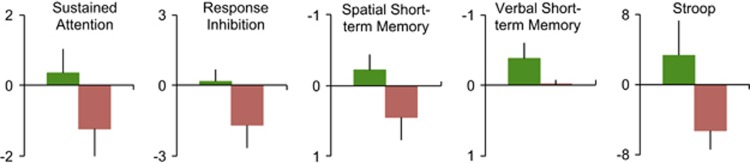
Change in cognitive performance from baseline to post-intervention (post – pre change) in the ONTRAC (*n*=11, in green) and active control group (*n*=7, in red). Data are averages, error bars are s.e.m. For all measures, improvement from baseline is shown above zero vs decline below zero. Performance changes for sustained attention and response inhibition were measured by signal detection sensitivity (*d*′). For spatial and verbal short-term memory, performance changes are shown for response speed in seconds (faster speeds at the post-assessment yield negative change from baseline and reflect improvement). Change in the Stroop test was as per the Stroop interference score. Post–pre intervention change in ONTRAC completers significantly differed from controls for response inhibition and Stroop (*P*⩽0.05) but not attention and short-term memory (*P*<0.14). ONTRAC, Online Neuroplasticity Targeted Remediation of Attention deficits in Children.

To assess changes in cognitive function, participants completed standard computerized tests of cognition at baseline and post-intervention. For this, we chose tests that measured sustained attention, response inhibition (non-impulsivity), visuospatial and verbal short-term memory and Stroop interference. All tests were different in content and task mechanics from the exercises presented in the ONTRAC program and in the control games, and we hypothesized that performance on these tests will benefit from the improved signal-to-noise resolution imparted by the ONTRAC training. Baseline to post-intervention changes in performance metrics for all five tests were submitted to a repeated-measures ANOVA with intervention arm (ONTRAC completers vs active controls) and assessment type as factors. This ANOVA showed an effect of intervention arm (F(1,16)=5.98, *P*=0.026) and an assessment × intervention arm interaction (F(4,64)=3.87, *P*=0.007). Between-arm results for each assessment were further parsed using two-tailed *t*-tests, showing *P*⩽0.05 significance in ONTRAC vs control post–pre change comparisons for the tests of response inhibition and Stroop interference. Improvement trends were also observed for tests of sustained attention, visuospatial and verbal short-term memory in the ONTRAC group but did not reach between-group significance (all *P*<0.14; Figure 4).

### Correlation between ONTRAC distractor training and ADHD symptoms

**Figure 5:**
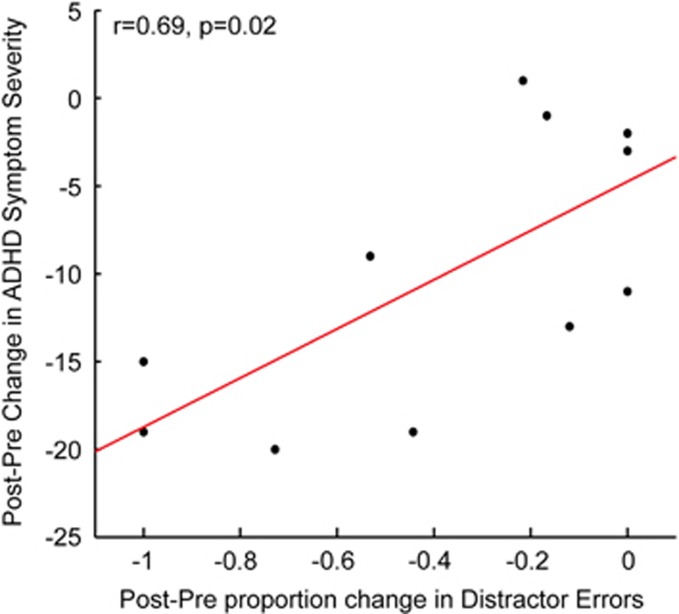
Correlation between improvements in distractor processing (*x* axis) trained by ONTRAC (*n*=11 completers) and improvements in ADHD symptom severity (*y* axis). More negative post–pre changes reflect larger improvement for both measures. ADHD, attention deficit/hyperactivity disorder; ONTRAC, Online Neuroplasticity Targeted Remediation of Attention deficits in Children.

Finally, we explored whether the novel component of ONTRAC, that is, distractor training, directly relates to the primary outcome measure, that is, improvements in ADHD symptom severity in children who completed ONTRAC. For this, we used visual distractor-suppression assessments embedded in the ONTRAC program, conducted at the beginning and end of the 30 h training period. We observed that the reduction in distractor-related errors (false positives) from beginning to end of ONTRAC training significantly correlated with the primary outcome measure, that is, post–pre improvements in ADHD symptom severity (*R*=0.69, *P*=0.02, Figure 5). Notably, only training-driven improvements in distractor suppression, but not improvements in attended-signal accuracy (*R*=0.20, *P*=0.55), correlated with ADHD symptom improvement.

## Discussion

This study was conducted as a global mental health collaboration between translational neuroscience and psychiatry research in the United States and India. An online cognitive training program, ONTRAC, was developed to target improvement in signal-to-noise resolution of sensory processing in children with ADHD. ONTRAC's targeted and personalized training approach focuses on enhancement of goal-relevant attended signals and suppression of sensory distractions.^17, 18, 25, 38^ We applied ONTRAC in Indian children with ADHD in a double-blind randomized controlled trial (RCT), which incorporated in-clinic assessments and at-home training. The ONTRAC and control group were assigned 30 h of training over 6 months; the control group played engaging video games that were also attention-demanding but that did not specifically target neuroplasticity-based improvement of sensory signal-to-noise resolution. The study outcomes evaluated the feasibility and initial efficacy of ONTRAC.

We showed that ONTRAC was feasible to deploy in Indian homes. Fifty-two percent of the children assigned to the ONTRAC-arm completed the 30 h program over 6 months, and the remaining children partially adhered. We did not find any significant differences between the training completers and the partial trainees in our feasibility assessments that specifically asked targeted questions about the training experience (enjoyment, frustration, satisfaction from training, ease of initiation, comprehension and navigation through training, therapeutic perception of training and so on). The families brought up factors unrelated to ONTRAC (technical issues such as sporadic Internet access and/or unexpected family stressors during the trial period) as influencing training adherence, while study personnel performing weekly check-ins observed that training completers in contrast to non-completers possibly had more supportive family environments (that is, parents showing greater interest in the child's progress through training). We further discuss this caveat in the ‘Limitations, challenges and best practices' section below.

In this initial trial, our primary efficacy outcome was parent-reported ADHD symptom severity. Significant improvement in ADHD symptoms was observed in the ONTRAC completers but not in the partial trainees or the control group. The intervention effect size (Cohen's *d*) was 0.31 at post-intervention and 0.36 at follow-up. These effect sizes are moderate^41^ but in the clinically meaningful range for psychiatric disorders. Moreover, sustained improvement at the follow-up visit 6 months beyond the training period is an important outcome that is not often observed even for standard-of-care pharmacological treatments.^11, 12, 13^

Additionally, we performed cognitive assessments at baseline and post-intervention and showed a significant overall difference in post vs pre change scores for the ONTRAC completers vs the control group. Further analyses revealed that the ONTRAC vs control group significantly differed on response inhibition and Stroop interference test outcomes, while group differences on sustained attention and short-term memory outcomes did not reach significance. Finally, a significant positive correlation emerged between the improvement in distractor suppression specifically trained by ONTRAC and the gains on the primary outcome measure, that is, improved ADHD behavioral symptoms. This correlation underscores the importance of the novel distractor training component of ONTRAC.

Overall, this initial trial is significant in several respects. It introduces use of cognitive therapeutics in low-to-middle-income country homes coupled with remote performance monitoring and periodic clinic assessments. Access to scalable neurotechnology in low-to-middle-income countries can be very useful, where mental health-care resources are sparse.^43, 44^ An at-home non-pharmacological training program is also agreeable with families in these settings where mental illness is especially stigmatized.^45^ Beyond demonstrating program feasibility, we also show some initial evidence for benefit in a double-blind RCT study. This is important as several commercial cognitive training programs claim benefits for children with attention deficits but have either not been tested in blinded, controlled trials, or do not show efficacy in such trials.^14, 15^ We also show evidence for sustained benefits, that is, reduced ADHD symptom severity for ONTRAC completers, at the follow-up visit, which aligns with our hypothesis that training core neural networks to enhance signal-to-noise resolution of information processing will benefit the ADHD child. We speculate that trained children possibly exercise and capitalize on their improved neurocognitive functions in real-world operations beyond the intervention environment, and hence demonstrate sustained gains.

### Limitations, challenges and best practices

Although the results of this initial study are encouraging, they must be interpreted with caution and cannot be generalized without replication in a larger RCT. The main limitation of our study is the low sample size related to practical issues with clinical recruitment in a low-to-middle-income country setting; stigma associated with psychiatric diagnoses in these settings deters patients and families from seeking clinical intervention and thus contributes to lower recruitment rates.^45^ Given the preliminary effect sizes observed here, the study will need replication in a trial with a 10 times larger sample for robustly powered effects.

The analyses in our study are further limited by the partial adherence observed in the ONTRAC group. Although targeted questions regarding training feasibility did not reveal significant differences between the ONTRAC completers and partial trainees, we acknowledge that inherent differences in participating families, especially the family support environment as observed by study personnel who tracked training, may affect adherence. This factor needs to be systematically assessed in future trials using appropriate scales (for example, the Family Environment Scale^46^) so that its interaction with intervention outcomes can be better understood. We also note that in this, to the best of our knowledge, first exploratory study we exclusively contrasted ONTRAC vs control participants who completed 30 h of intervention in all analyses, as we hypothesized that partaking in only a few training hours will not adequately stimulate neuroplasticity mechanisms and hence show no clinical benefit. Intent-to-treat analyses were not followed in this initial study, that is, analyses that include data for partial adherents and dropouts, but a future well-powered trial needs to conform to intent-to-treat standards. Finally, ADHD symptom ratings obtained from multiple sources, parents, teachers and the blinded clinical assessors would strengthen findings in a future trial.

Here we also note challenges related to double blinding in a cognitive training RCT. Although there is no doubt that this standard has to be followed to claim any benefits of an experimental intervention, the choice of control needs to be carefully considered as it may influence blinding. Ideally, the control games should be balanced in all aspects of fun, variety, complexity and novelty with the experimental game-based intervention. Practically this is hard to achieve, for example, here we used commercial video games that inherently had a greater element of fun; children confirmed them to be more enjoyable in the feasibility surveys and the control group had greater adherence rates than the ONTRAC group. Although ratings of perceived therapeutic benefit were the same in ONTRAC completers vs control children, we did not assess whether the ONTRAC vs control games differentially influenced parental perception of therapeutic benefit, which could be the case. This may be important to assess in a future study as parents provide the ADHD symptom ratings. Notably, we did try to minimize expectation bias as families were not aware that another computerized training program other than their assigned arm was also being tested. Multiple converging sources of symptom reporting, teachers and clinicians in addition to parents, should also help verify successful blinding in a future study. In this context, some studies have used a non-performance-adaptive version of the experimental training as a control.^47, 48, 49^ As this control presents an identical user-facing front-end as the actual training, it could be argued to preserve blinding better. But this non-adaptive control can become so boring and repetitive, especially over long training periods, 6-month duration in our study, that adherence especially in the remotely monitored home setting becomes problematic. Overall, when choosing a control for a field study, researchers need to find the right balance between what will be feasible to implement and also be well-matched to the experimental training so that double blinding is effectively maintained within participating families and clinical assessors. In our opinion and that of others,^40, 50, 51^ control games do serve as an effective control; nevertheless, blinding in a future trial would further benefit if we are able to match the elements of fun, variety and novelty between the experimental and control trainings even more closely.

In summary, much further research remains to be done to rigorously show whether a neuroplasticity-targeted computerized training program such as ONTRAC can become an accessible, scalable and validated treatment option for children limited by ADHD. This study is a first step in the right direction implementing best practices in its double-blind RCT study design and multiple assessment time points including a 6-month follow-up. Yet, as we have discussed, future work needs to perfect this design in its high-powered sample size combined with assessments of family environment, multiple sources of symptom assessments, an even more carefully matched control and integration of intent-to-treat analyses.

## Figures and Tables

**Table 1:** Results of the feasibility survey in ONTRAC completers (*n*=11), partial trainees (*n*=10) and the active control group (*n*=7)

*Feasibility survey*	*ONTRAC completers*	*ONTRAC partial trainees*	*Active control*
I enjoyed the training	5.0±0.6	3.7±0.7	5.3±0.6
I felt frustrated after training	2.8±0.6	2.8±0.5	2.5±0.8
I felt satisfied after training	5.0±0.6	4.7±0.8	4.8±0.7
I felt tired after training	4.1±0.7	3.3±0.7	3.0±0.8
The program was easy to understand	5.7±0.5	4.7±0.7	4.5±0.9
The program was difficult to use	3.1±0.6	4.7±0.7	2.7±0.8
The program was easy to navigate	5.1±0.6	3.2±0.6	4.8±1.0
I was worried about my data security	2.8±0.6	4.2±0.8	2.3±0.8
The program was easy to initiate each day	4.5±0.8	3.8±0.9	4.7±1.0
The program graphics were attractive	3.8±0.6	3.3±0.9	5.5±0.6
The program easily fit in my daily schedule	4.9±0.6	3.5±0.8	2.5±0.6
The training session passed by quickly	5.2±0.5	4.0±0.8	4.3±0.8
The training felt therapeutic to me	4.6±0.6	3.8±0.7	4.0±0.7
The training felt useless to me	3.6±0.6	4.5±0.3	3.2±0.8
I would use this training outside of this study	3.8±0.6	3.0±0.5	3.0±0.9
I would recommend this training to others	4.6±0.7	3.5±0.8	5.2±1.0
This training has positively affected my life	4.7±0.5	4.0±0.7	4.8±0.3

Abbreviation: ONTRAC, Online Neuroplasticity Targeted Remediation of Attention deficits in Children.

Data are mean±s.e.m. on seven-point Likert scale assessments. ONTRAC completers and active control participants differed on enjoyment, frustration and ease of program initiation (all *P*<0.04); there were no other significant group differences.
